# Autophagy protein NRBF2 attenuates endoplasmic reticulum stress-associated neuroinflammation and oxidative stress via promoting autophagosome maturation by interacting with Rab7 after SAH

**DOI:** 10.1186/s12974-021-02270-4

**Published:** 2021-09-16

**Authors:** Hanhai Zeng, Huaijun Chen, Min Li, Jianfeng Zhuang, Yucong Peng, Hang Zhou, Chaoran Xu, Qian Yu, Xiongjie Fu, Shenglong Cao, Jing Cai, Feng Yan, Gao Chen

**Affiliations:** 1 Department of Neurological Surgery, The Second Affiliated Hospital of Zhejiang University School of Medicine, Jiefang Road 88th, Hangzhou, 310009 Zhejiang Province China; 2 Neurosurgical Intensive Care Unit, The Second Affiliated Hospital of Zhejiang University School of Medicine, Jiefang Road 88th, Hangzhou, 310009 Zhejiang Province China

**Keywords:** Subarachnoid hemorrhage, NRBF2, Rab7, Autophagy maturation, Endoplasmic reticulum stress, Neuroinflammation, Oxidative stress

## Abstract

**Background:**

Neuroinflammation and oxidative stress plays an important role in the pathogenesis of early brain injury (EBI) after subarachnoid hemorrhage (SAH). This study is the first to show that activation of autophagy protein nuclear receptor binding factor 2 (NRBF2) could reduce endoplasmic reticulum stress (ERS)-associated inflammation and oxidative stress after SAH.

**Methods:**

Male C57BL/6J mice were subjected to endovascular perforation to establish a model of SAH. NRBF2 overexpression adeno-associated virus (AAV), NRBF2 small interfering RNAs (siRNA), lysosomal inhibitor-chloroquine (CQ), and late endosome GTPase Rab7 receptor antagonist-CID1067700 (CID) were used to investigate the role of NRBF2 in EBI after SAH. Neurological tests, brain water content, western blotting and immunofluorescence staining were evaluated.

**Results:**

Our study found that the level of NRBF2 was increased after SAH and peaked at 24 h after SAH. In addition, we found that the overexpression of NRBF2 significantly improved neurobehavioral scores and reduced ERS, oxidative stress, and neuroinflammation in SAH, whereas the inhibition of NRBF2 exacerbated these phenotypes. In terms of mechanism, NRBF2 overexpression significantly promoted autophagosome maturation, with the downregulation of CHOP, Romo-1, TXNIP, NLRP3, TNF-α, and IL-1β expression through interaction with Rab7. The protective effect of NRBF2 on ERS-associated neuroinflammation and oxidative stress after SAH was eliminated by treatment with CQ. Meanwhile, it was also reversed by intraperitoneal injection of CID. Moreover, the MIT domain of NRBF2 was identified as a critical binding site that interacts with Rab7 and thereby promotes autophagosome maturation.

**Conclusion:**

Our data provide evidence that the autophagy protein NRBF2 has a protective effect on endoplasmic reticulum stress-associated neuroinflammation and oxidative stress by promoting autophagosome maturation through interactions with Rab7 after SAH.

**Supplementary Information:**

The online version contains supplementary material available at 10.1186/s12974-021-02270-4.

## Introduction

Subarachnoid hemorrhage (SAH), a subtype of stroke, is caused mainly by aneurysm rupture, resulting in poor neurological deficits, and high morbidity and mortality [1, 2]. The underlying mechanisms that cause brain injury after SAH include increased endoplasmic reticulum stress (ERS), oxidative stress injury, neuroinflammation, and neuronal apoptosis [3–5]. More and more studies support the view that ERS plays an important role in the pathophysiological process after SAH [3, 6, 7]. The excessive activation of ERS induces oxidative stress, and then triggers downstream cascade reactions, leading to inflammation [8]. The activation of the thioredoxin-interacting protein (TXNIP)/NOD-like receptor pyrin domain-containing 3 protein (NLRP3) inflammasome can link ERS to neuroinflammation in brain tissues [6, 7].

Nuclear receptor binding factor 2 (NRBF2), a regulatory subunit of the ATG14-BECN1/Beclin 1-PIK3C3 complex, is involved in multiple diseases and stress conditions [9–11]. Importantly, NRBF2 modulates autophagosome formation by regulating ATG14-linked PIK3C3 activity for autophagosome biogenesis [9–12]. Lu et al*.* reported that NRBF2 modulates autophagy via regulation of PI3K-III and prevents ERS-mediated cytotoxicity and liver injury [9]. In addition, studies of NRBF2 in Alzheimer’s disease revealed that NRBF2 plays an important role in regulating the degradation of APP C-terminal fragments by modulating autophagy and could be a potential therapeutic target for Alzheimer’s disease [11, 13]. Many factors regulate the fusion of autophagosomes with late endosomes/lysosomes. A recent study on intestinal inflammation indicated that NRBF2 was vital for the generation of an activated subtype of Rab7 to promote the fusion between phagosomes and lysosomes by interacting with the MON1-CCZ1 complex [10]. Additionally, Cai et al. revealed a required role of NRBF2 in modulating autophagosome maturation by interacting with Rab7 and Alzheimer disease-associated protein degradation [14]. In addition, the activation of autophagy could suppress the overactivation of ERS and reduce neuroinflammation [15]. However, the mechanisms underlying how NRBF2 regulates ERS and suppresses neuroinflammation after SAH has not been thoroughly explored.

Here, (i) we identified the increased level of NRBF2 after SAH and the protective effect of NRBF2 on the modulation of ERS-associated neuroinflammation and oxidative stress. (ii) We further found that the protective effect of NRBF2 occurred through enhancing autophagosome maturation by interacting with Rab7. (iii) Finally, we demonstrated that the MIT domain of NRBF2 was necessary for the interaction between NRBF2 and Rab7.

## Materials and methods

### Animal models of SAH

This study involving animals was in accordance with the Guide for the Care and Use of Laboratory Animals published by the National Institutes of Health. All experimental protocols were approved and supervised by the Institutional Animal Care and Use Committee of Zhejiang University.

Adult male C57BL/6 mice (approximately 8 weeks) weighing 22–25 g, obtained from SLAC Laboratory Animal Company (Shanghai, China), were used to induce SAH by endovascular perforation according to the methods of the previous study [1, 16]. Briefly, after exposing the left carotid artery and its branches, a 5–0 sharpened monofilament nylon suture was advanced and finally reached the bifurcation of the anterior and middle cerebral artery. Then, vessel perforation was executed to produce SAH (Supplementary Text S1). The same procedure was performed in the sham group, except for blood vessel wall puncture.

### Experimental group design (Supplemental Fig. 1)

#### Experiment 1

Mice were randomly assigned to six groups including the sham group, 6 h after SAH group, 12 h after SAH group, 24 h after SAH group, 48 h after SAH group, and 72 h after SAH group for western blotting detection of NRBF2 expression changes. In addition, mice were randomly distributed to sham and SAH-24 h group for the cellular co-localization of NRBF2 examined by double-labeled fluorescent staining, and qualitative expression of NRBF2 examined by immunohistochemical (IHC) staining.

#### Experiment 2

Mice were randomly assigned into six groups, including the sham, SAH, SAH + Scramble (Scr)-siRNA, SAH + NRBF2-siRNA, SAH + negative control (NC)-AAV, SAH + NRBF2-AAV groups, for the assessment of SAH grading score, neurological function (for 24 h, 72 h, and long-term neurological function evaluation after SAH), brain water content (BWC), western blotting, immunofluorescence (IF) staining, and dihydroethidium (DHE) staining. In addition, the cellular co-localization of NRBF2 and LAMP2 was examined by double-labeled fluorescent staining in SAH.

#### Experiment 3.1

Mice were randomly assigned into five groups, including the sham, SAH + NC-AAV, SAH + NRBF2-AAV, SAH + NRBF2-AAV + vehicle, SAH + NRBF2-AAV + 3-methyladenine (3-MA) groups, for the assessment of SAH grading score, neurological function, western blotting, and BWC.

#### Experiment 3.2

Mice were randomly assigned into the five groups including the sham, SAH + Scr-siRNA, SAH + NRBF2-siRNA, SAH + NRBF2-siRNA + vehicle, SAH + NRBF2-siRNA + rapamycin (Rapa) groups, for the assessment of SAH grading score, neurological function, western blotting, and BWC.

#### Experiment 4

Mice were randomly assigned into five groups, including the sham, SAH + NC-AAV, SAH + NRBF2-AAV, SAH + NRBF2-AAV + vehicle, and SAH + NRBF2-AAV + chloroquine (CQ) groups, for the assessment of SAH grading score, neurological function, BWC, and western blotting.

#### Experiment 5.1

Mice were randomly assigned to two groups, the sham and SAH groups, for co-immunoprecipitation (Co-IP). Moreover, the cellular co-localization of NRBF2 and Rab7 was examined by double-labeled fluorescent staining in SAH.

#### Experiment 5.2

Mice were randomly assigned into five groups, including the sham, SAH + NC-AAV, SAH + NRBF2-AAV, SAH + NRBF2-AAV + vehicle, and SAH + NRBF2-AAV + CID1067700 (CID) groups, for the assessment of SAH grading score, neurological function, BWC, western blotting, and IF staining.

#### Experiment 6.1

HT22 cells were randomly assigned into two groups, the control and Hemin groups, for the assessment of co-IP. Moreover, the cellular co-localization of NRBF2 and Rab7 was examined by double-labeled fluorescent staining in the Hemin group.

#### Experiment 6.2

HT22 cells were randomly assigned into three groups: the Hemin + Flag-plasmid, Hemin + NRBF2-plasmid, and Hemin + ΔCARD-plasmid for the assessment of co-IP.

### Drug administration

NRBF2 siRNA and scramble siRNA were infused into the right lateral ventricle at 24 h before SAH induction. NRBF2-AAV (1.78e + 13 vg/ml, 3 μl) or scrambled NC-AAV (2.54e + 12 vg/ml, 3 μl) obtained from Shanghai Genechem Co., Ltd. was infused into the right lateral ventricle at 3 weeks before SAH induction (Supplementary Text S1). 3-MA (7.5 μg dissolved in normal saline by heating the solution to 60–70 °C immediately before injection) or vehicle (normal saline) was administered by intracerebroventricular injection at the onset of SAH [17]. Rapa (25 μM dissolved in 2% DMSO, 2 μl) or vehicle (2% DMSO) was administered by intracerebroventricular injection 30 min after SAH induction [18]. CID (2.5 mg/kg dissolved in 2% DMSO) or vehicle (2% DMSO) was administered intraperitoneally 10 min before and 90 min after the onset of SAH [19]. CQ (60 mg/kg dissolved in normal saline) or vehicle (normal saline) was administered intraperitoneally immediately after SAH [20].

### SAH grade

The 18-point SAH severity grading system was used at 24 h after SAH as previously reported [5]. The basal part of the mouse brain was divided into six parts and each part was blindly evaluated on a scale of 0–3 judging by the amount of the subarachnoid clot.

### Neurological score evaluation

The modified Garcia scoring system [21, 22] (Supplemental Table S1) and Behavior score [23] (Supplemental Table S2) were blindly assessed for neurological function at 24 h and 72 h after SAH. The total score ranged from 3 to 18 for the modified Garcia scoring system and the total score ranged from 0 to 6 for the behavior score. Higher scores for the modified Garcia scoring system and lower scores for the behavior score indicated a better neurological function.

Moreover, the Morris water maze (MWM) test was used to test long-term neurological impairments from 22 to 28 days post-SAH as previously described [23]. First, mice were trained to find the escape platform. Second, the mice were trained for 5 consecutive days. Third, mice received a probe trial, in which the platform was removed, and then the escape latency, swimming distance, platform crossovers, and time spent in the target quadrant were tracked and analyzed by the SMART software (Panlab, USA).

### Brain water content (BWC)

BWC was calculated to evaluate the severity of brain edema [1]. After sacrificed, mouse brains were rapidly removed and divided into the left brain, right brain, cerebellum, and brain stem at 24 h post-SAH. Each part was directly weighed to obtain the wet weight and then dried at 105 °C for 72 h to obtain the dry weight. The BWC was calculated as [(wet weight−dry weight)/wet weight] × 100%.

### Cell culture and transfection

HT22 cells were unfrozen and incubated with DMEM medium with 10% fetal bovine serum (FBS) offered by Thermo Fisher (USA) at 37 °C and 5% CO2. And HT22 cells were stimulated with 200 μM hemin (Sigma-Aldrich, MO, USA, Cat. No. H9039) to induce the SAH model in vitro [24]. Overexpression of NRBF2 was induced with plasmid and performed using Lipofectamine 3000 reagent (Thermo Fisher, USA). Efficiency of transfection was detected by RT-qPCR at 48 h.

### Immunohistochemistry and immunofluorescence staining

IHC and IF staining were conducted as previously described [23, 25]. For IHC, brain sections (8-μm thickness) were immersed in acetone for 24 h and then incubated with 3% hydrogen peroxide to eliminate endogenous peroxidase activity. After blocking with 5% bovine serum albumin (BSA) and 0.3% Triton X-100, sections were incubated with rabbit anti-NRBF2 (1:100, Proteintech, 24858-1-AP) overnight at 4 °C. Sections were incubated with secondary antibodies for 1 h at 37 °C and then visualized with a 3,3-diaminobenzidine (DAB) for 2 min. Images were observed under a light microscope (Leica, Mannheim, Germany). For IF, brain sections (8-μm thickness) or cell climbing slices were blocked with 5% bovine serum albumin (BSA) and 0.3% Triton X-100 for 2 h at room temperature. Sections were incubated overnight at 4 °C with rabbit anti-NRBF2 (1:100, Proteintech, 24858-1-AP), mouse anti-NeuN (1:500, Abcam, ab104224), mouse anti-GFAP protein (1:500, Abcam, ab10062), goat anti-Iba-1 (1:500, Abcam, ab-5076), rabbit anti-LC3B (1:200, CST, #3868), mouse anti-Lamp2 (1:100, Proteintech, 66301-1-Ig), rabbit anti-Lamp2 (1:100, Proteintech, 27823-1-AP), rabbit anti-CHOP (1:100, Proteintech, 15204-1-AP), and mouse anti-Rab7 (1:200, CST, #95746). Sections were incubated with secondary antibodies for 2 h at room temperature. Images were observed by a fluorescence microscope (Leica, Mannheim, Germany). The results were analyzed by ImageJ software.

### Western blotting

Western blotting was performed as previously described [26]. Proteins from samples were lysed with RIPA lysis buffer. Forty micrograms of protein was separated by 7.5–15% SDS-PAGE. Then, the protein transferred to PVDF membranes. The PVDF membranes were blocked in 5% skim milk for 1 h at room temperature and incubated overnight at 4 °C with the following primary antibodies: rabbit anti-NRBF2 (1:1000, CST, #8633), rabbit anti-LC3B (1:1000, CST, #2775), mouse anti-Lamp2 (1:1000, Abcam, 203224), rabbit anti-Romo1 (1:200, AVIVA Systems Biology, ARP58431_P050), rabbit anti-GRP78 (1:1000, Proteintech, 11587-1-AP), rabbit anti-CHOP (1:1000, Proteintech, 15204-1-AP), rabbit anti-TXNIP (1:1000, Abcam, 188865), rabbit anti-NLRP3 (1:1000, Abcam, 263899), rabbit anti-TNF-α (1:1000, CST, #11948), rabbit anti-IL-1β (1:1000, CST, #31203), mouse anti-Rab7 (1:1000, CST, #95746), rabbit anti-CCZ1 (1:1000, Proteintech, 22159-1-AP), and goat anti-Mon1A (1:200, Novus Biologicals, NBP1-52007). The PVDF membranes were incubated with horseradish peroxidase-conjugated secondary antibodies for 1 h at room temperature. The blots were visualized using the ECL Plus chemiluminescence reagent kit (Amersham Bioscience, Arlington Heights, IL, USA), and protein quantification was performed by ImageJ.

### Co-IP detection

Co-IP was conducted as previously described [27]. Samples were lysed and extracted followed by centrifugation. Protein (500 μg) was incubated with rabbit anti-NRBF2 (5 μg, CST, #8633) or control IgG overnight at 4 °C. The immune complexes were then linked to protein A/G-agarose beads for 4 h. The eluted proteins were loaded onto SDS-PAGE gels.

### Measurement of ROS level

Dihydroethidium (DHE) staining [5] was used to measure the oxidative stress level of mouse brains. For DHE staining, freshly prepared frozen brain slices (8 μm) were incubated with 2 μmol/L fluorescent dye dihydroethidium (DHE, Thermo Fisher Scientific, USA) at 37 °C for 30 min in a humidified chamber and protected from light. Images of DHE staining were obtained and the red fluorescence intensity was quantified by using ImageJ software.

### Statistical analysis

Continuous data are showed as the mean ± standard deviation (SD) or median (interquartile range) based on the normality and homogeneity of variance. For the data with a normal distribution, significant differences among groups were analyzed using Student’s *t* test (2 groups) and one-way analysis of variance (ANOVA) (≥ 3 groups). For the data that failed to be normally distributed, significant differences among groups were analyzed using the Mann-Whitney *U* test (2 groups) or Kruskal-Wallis test (≥ 3 groups). A *P* value less than 0.05 indicated statistical significance. GraphPad Prism and SPSS software (Version 23.0) were used for statistical analyses. Investigators were blinded to the identity of groups during the whole experiment.

## Results

### SAH model

Physiological parameters, including mean arterial pressure, arterial pH, PO2, PCO2, and blood glucose levels, were monitored during the surgical procedure. No significant changes in those physiological variables were noted among the different groups (Supplementary Table S3).

### Time-course expression of NRBF2 after SAH

**Fig. 1:**
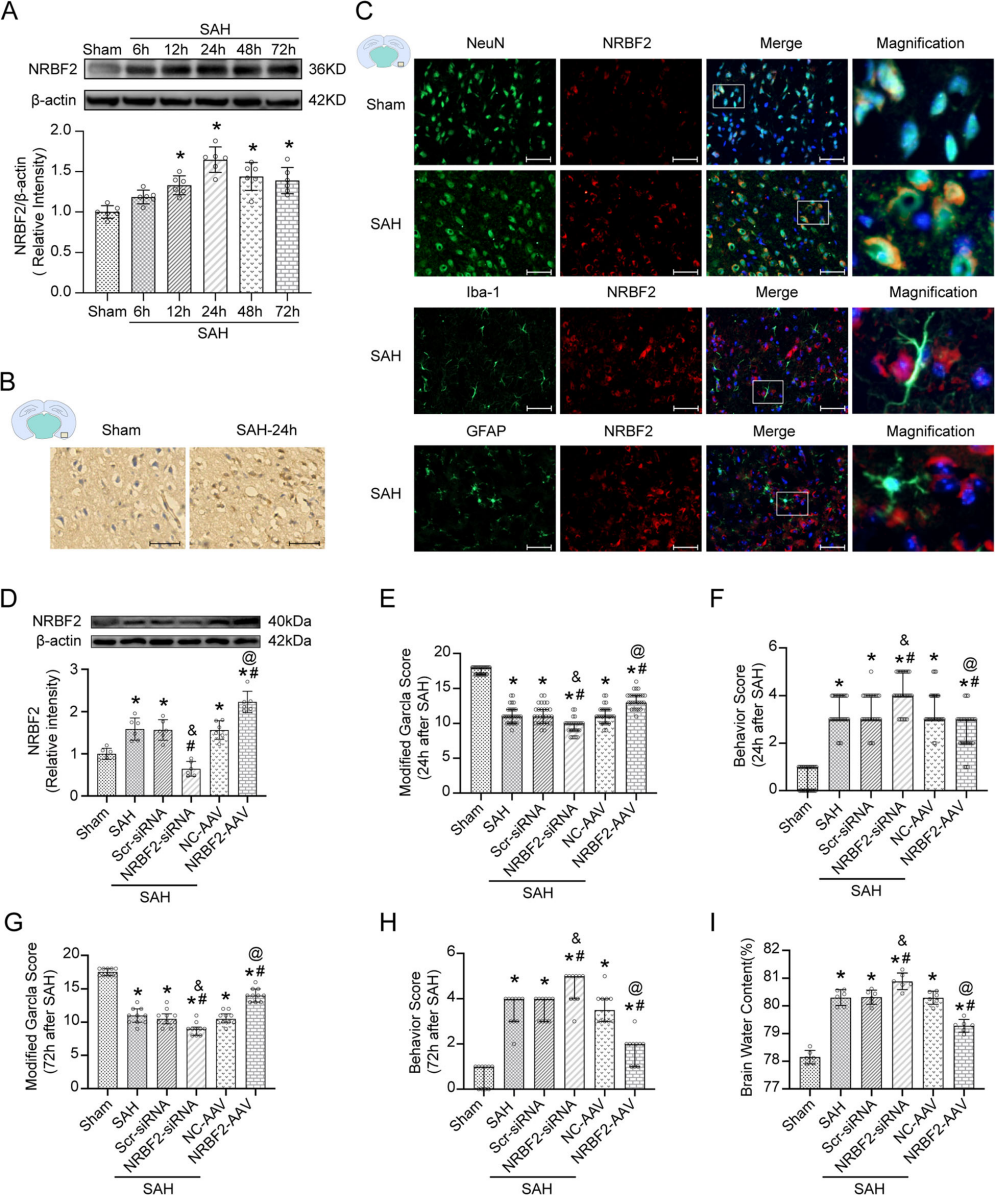
Expression, distribution, and effect of NRBF2 after SAH. **A** Representative western blotting images and quantitative analyses of NRBF2 expression in ipsilateral basal cortex after SAH. *n* = 6. **B** Representative microphotographs of immunohistochemistry staining showing the expression of NRBF2 in sham group and SAH 24 h group. *n* = 3. **C** Representative microphotographs of immunofluorescence double staining showing the localization of NRBF2 (red) with NeuN, GFAP, and Iba-1 (green) in sham group and SAH 24 h group. *n* = 3. **D** Representative picture and quantitative analysis of NRBF2 in different groups. *n* = 6. **E**–**H** Quantification of neurological function with two different scoring systems at 24 h (*n* = 27) and 72 h (*n* = 10) after SAH. **I** Quantification of brain water content. *n* = 6. Data are represented as mean ± SD (**A**, **D**, **I**) or median (interquartile range) (**E**–**H**). Scale bar = 50 μm. *P < 0.05 versus sham group. #*P* < 0.05 versus SAH group. &*P* < 0.05 versus SAH + Scr-siRNA group. @*P* < 0.05 versus SAH + NC-AAV group

Western blot results indicated that the level of NRBF2 was increased after SAH and peaked at 24 h (Fig. 1A). Consistently, IHC and IF confirmed the increased expression of NRBF2 at 24 h after SAH. Moreover, double IF staining showed that NRBF2 was mainly located in neurons (NeuN) of the cerebral cortex rather than microglia or astrocytes (Fig. 1B, C).

### Effect of NRBF2 on short-term and long-term neurological function after SAH

To investigate the potential role of NRBF2 in the pathological process after SAH, downregulation and upregulation of NRBF2 were performed with NRBF2 siRNA and NRBF2 overexpression mediated by adeno-associated virus (AAV). Western blotting analysis showing the increased level of NRBF2 with AAV-mediated overexpression and decreased expression of NRBF2 with siRNA (Fig. 1D).

When the mice were sacrificed and brain samples were collected, no significant difference in SAH grade among the modeling groups was noted (Supplementary Fig. S2B). We investigated the effectiveness of NRBF2 regulation on aggravated neurological deficits evaluated by the modified Garcia and Behavior score and cerebral edema due to SAH. Neurobehavioral scores were evaluated at both 24 h and 72 h after SAH. The data showed that upregulation of NRBF2 ameliorated the neurobehavioral scores and brain water content in the SAH + NRBF2-AAV group compared with those in the SAH + NC-AAV group, while downregulation of NRBF2 aggravated the neurobehavioral scores and brain water content (Fig. 1E–I).

The MWM was also introduced to evaluate the effect of NRBF2 on persistent cognitive impairment. Data from the MWM indicated no significant differences in escape latency and swimming distance was noted on day 1, suggesting that there were no significant differences in swimming ability or visual impairment that were comparable among animals at baseline (Supplementary Fig. S3A, B). However, in the following days from day 2 to day 5, mice in the SAH + NRBF2-siRNA group presented a worse performance with increased escape latency and longer swimming distance than those in the SAH + Scr-siRNA group (Supplementary Fig. S3A, B). However, mice in the SAH + NRBF2-AAV group manifested a better performance with decreased escape latency and shorter swimming distance than those in the SAH + NC-AAV group (Supplementary Fig. S3A, B). In addition, swimming trials suggested that mice in the SAH + NRBF2 siRNA group exhibited fewer crossovers and spent less time in the target quadrant in the MWM than that of mice in the SAH + Scr siRNA group. However, mice in the SAH + NRBF2-AAV group demonstrated a better performance in the target quadrant (Supplementary Fig. S3C–E).

### Effect of NRBF2 on autophagy, endoplasmic reticulum stress, oxidative stress, and neuroinflammation at 24 h after SAH

**Fig. 2:**
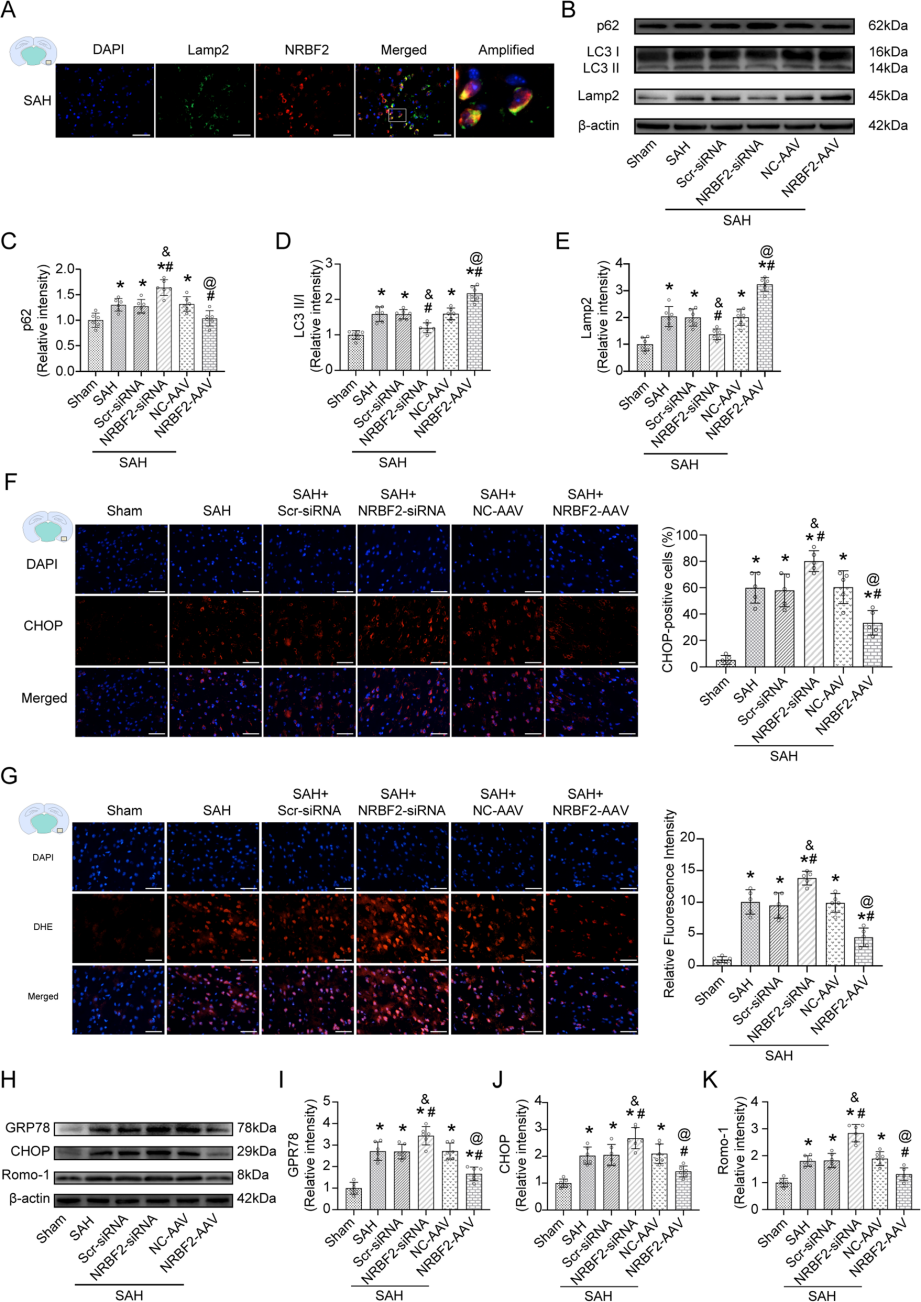
Effect of NRBF2 on autophagy, endoplasmic reticulum stress, and oxidative stress after SAH. **A** Representative microphotographs of immunofluorescence double staining showing the localization of NRBF2 (red) with Lamp2 (green) in SAH 24 h group. *n* = 3. **B**–**E** Representative western blotting images and quantitative analyses of p62, LC3 II/I, and Lamp2 expression at 24 h after SAH. *n* = 6. **F** Representative microphotographs and quantitative analysis of CHOP-positive cells. *n* = 5. **G** Representative microphotographs and quantitative analysis of relative fluorescence intensity of DHE (red). *n* = 5. **H**–**K** Representative western blotting images and quantitative analyses of GRP78, CHOP, Romo-1 expression at 24 h after SAH. *n* = 6. Scale bar = 50 μm. Data are represented as mean ± SD. *P < 0.05 versus sham group. #*P* < 0.05 versus SAH group. &*P* < 0.05 versus SAH + Scr-siRNA group. @*P* < 0.05 versus SAH + NC-AAV group

Double fluorescence showed that NRBF2 was co-localized with the lysosomal-associated membrane marker Lamp2 after SAH, suggesting that NRBF2 was related to the maturation of autophagosomes (Fig. 2A). Data from the western blotting analysis demonstrated that the expression levels of p62, LC3, and Lamp2, were remarkably higher in the SAH group than in the sham group. Meanwhile, NRBF2 siRNA injection further decreased the expression of LC3 and Lamp2, and increased the expression of p62, compared to the SAH + Scr siRNA group. Conversely, NRBF2 AAV injection further enhanced the expressions of LC3 and Lamp2, and decreased the expressions of p62, compared to the SAH + NC-AAV group (Fig. 2B–E). To further confirm whether NRBF2 exerts its protective effect against EBI after SAH by regulating autophagy, mice overexpressing NRBF2 were treated with the autophagy inhibitor 3-MA. We observed that efficient 3-MA treatment as evidenced by a decrease in autophagic flux (Supplementary Fig. S4A–C), significantly abolished the neuroprotective effects of NRBF2, presenting aggravated neurological deficits (Supplementary Fig. S4D, E) and cerebral edema (Supplementary Fig. S4F). In contrast, treatment with the autophagy inducer Rapa significantly abolished the neurological impairment of NRBF2 downregulation, as evidenced by an increase in autophagic flux (Supplementary Fig. S4G–I), with aggravated neurological deficits (Supplementary Fig. S4J, K) and cerebral edema (Supplementary Fig. S4L). These data demonstrated that autophagy was involved in the protective effect of NRBF2 against EBI after SAH.

Data from staining demonstrated that overexpression of NRBF2 evidently inhibited the increase in the number of CHOP-positive cells post-SAH, whereas knockdown of NRBF2 increased the number of CHOP-positive cells (Fig. 2F). Furthermore, western blotting analysis also demonstrated increased expression of the ERS-related protein GPR78 and CHOP in the SAH group compared with the sham group. Meanwhile, overexpression of NRBF2 by AAV further eminently counteracted the above-mentioned alterations, while knockdown of NRBF2 enhanced the expression of GPR78 and CHOP (Fig. 2H–J).

Data from DHE staining demonstrated that the levels of ROS were remarkably increased in the SAH group compared with the sham group. Meanwhile, knockdown of NRBF2 enhanced the production of ROS, while AAV-mediated overexpression of NRBF2 decreased the level of ROS (Fig. 2G). Moreover, in line with the results demonstrated by IF, similar changes in the oxidative stress protein Romo-1 from diverse groups were confirmed by western blot (Fig. 2H, K).

**Fig. 3:**
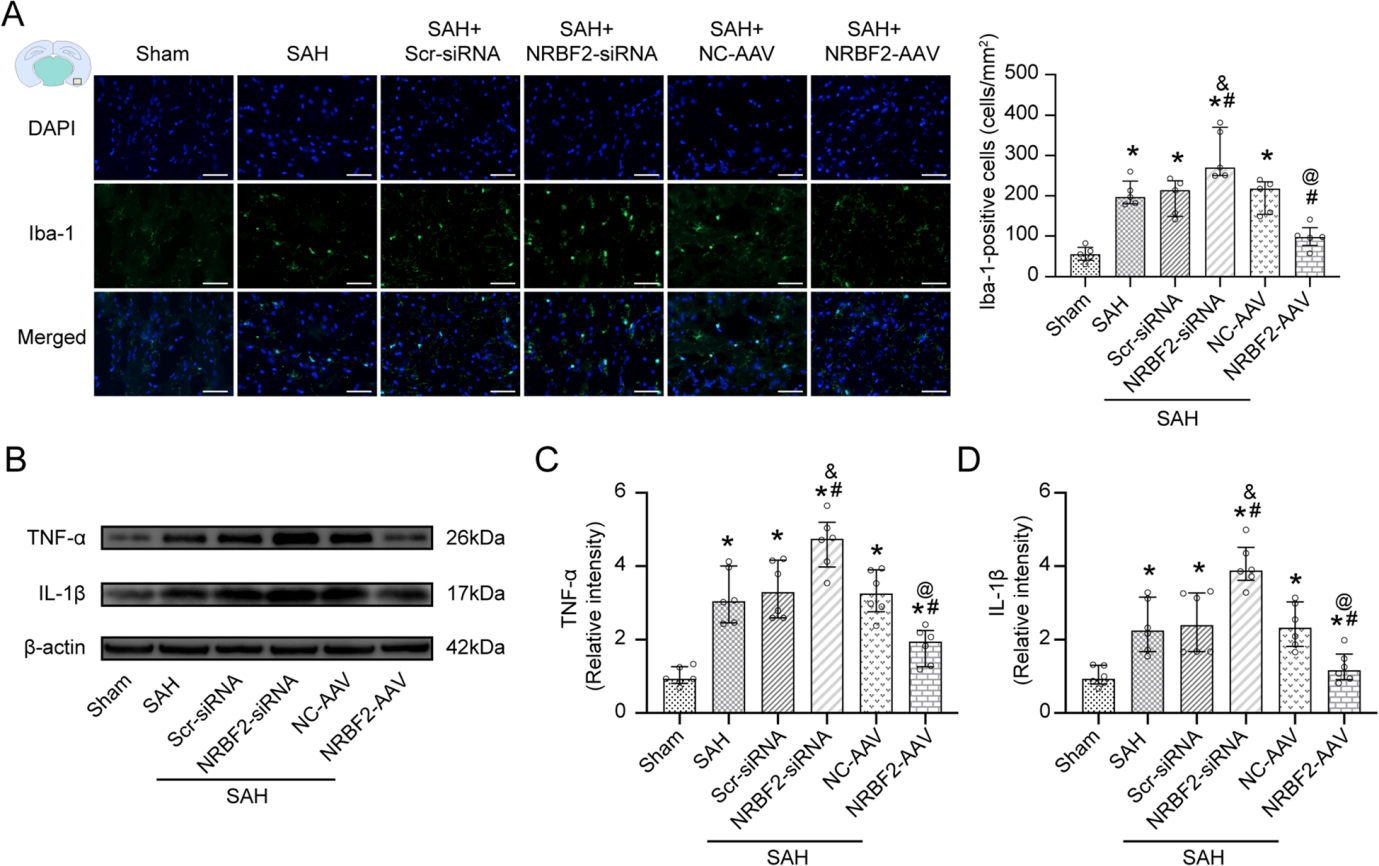
Effect of NRBF2 on neuroinflammation after SAH. **A** Representative microphotographs and quantitative analysis of Iba-1-positive cells. *n* = 5. Scale bar = 50 μm. **B**–**D** Representative western blotting images and quantitative analyses of TNF-α, and IL-1β expression at 24 h after SAH. *n* = 6. Data are represented as mean ± SD. *P < 0.05 versus sham group. #*P* < 0.05 versus SAH group. &*P* < 0.05 versus SAH + Scr-siRNA group. @*P* < 0.05 versus SAH+NC-AAV group

IF staining also showed that the overexpression of NRBF2 evidently inhibited the increase in the number of Iba-1 positive microglia post-SAH, whereas knockdown of NRBF2 increased the number of Iba-1 positive cells (Fig. 3A). Furthermore, western blotting analysis also demonstrated increased expression of the neuroinflammation-related protein TNF-α and IL-1β in the SAH group compared with the sham group. Meanwhile, overexpression of NRBF2 by AAV further eminently counteracted the above-mentioned alterations, while knockdown of NRBF2 enhanced the expression of TNF-α and IL-1β (Fig. 3B–D).

### Inhibition of autophagosome and lysosome fusion reversed the protective effects of NRBF2 on endoplasmic reticulum stress-associated neuroinflammation and oxidative stress

**Fig. 4:**
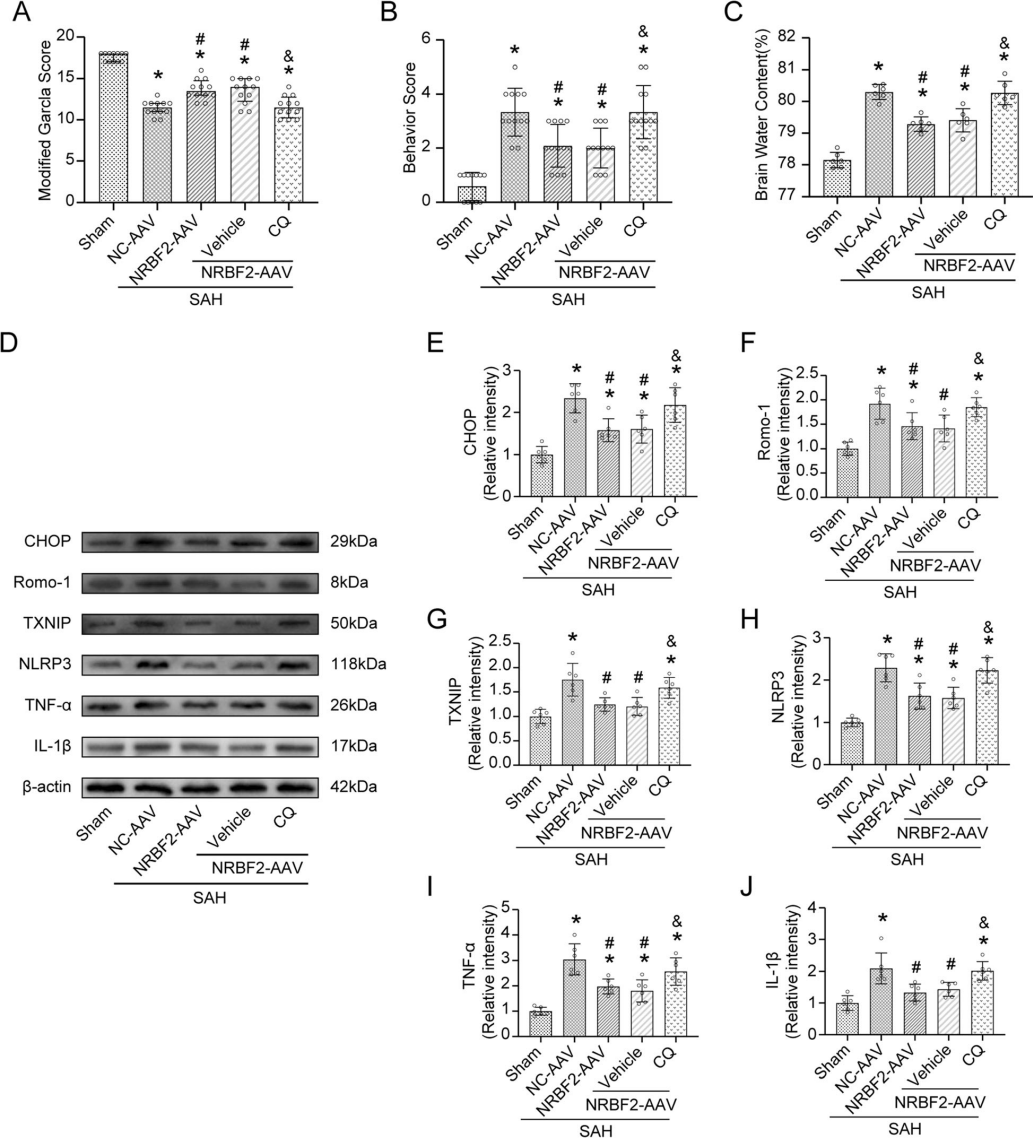
Pharmacological inhibition of the fusion of autophagosome and lysosome abolished the neuroprotective effect of NRBF2 against SAH at 24 h. **A**, **B** Quantification of neurological function with two different scoring systems at 24 h after SAH. *n* = 12. **C** Quantification of brain water content. *n* = 6. **D**–**J** Representative western blotting images and quantitative analyses of CHOP, Romo-1, TXNIP, NLRP3, TNF-α, and IL-1β expression at 24 h after SAH. *n* = 6. Data are represented as mean ± SD (**C**, **E**–**J**) or median (interquartile range) (**A**, **B**). *P < 0.05 versus sham group. #*P* < 0.05 versus SAH + NC-AAV group. &*P* < 0.05 versus SAH + NRBF2-AAV + vehicle group

Compared with the control groups (SAH + NC-AAV group), the group treated with the lysosomal inhibitor CQ abated the beneficial effect derived from NRBF2 overexpression on neurological scores and cerebral edema (Fig. 4A–C). Additionally, compared with the SAH + NC-AAV group, the expression of CHOP, Romo-1, TXNIP, NLRP3, TNF-α, and IL-1β were downregulated in the SAH + NRBF2-AAV group (Fig. 4D–J). Conversely, CQ injection terminated the beneficial effect derived from NRBF2 overexpression, resulting in increased expression of CHOP, Romo-1, TXNIP, NLRP3, TNF-α, and IL-1β (Fig. 4D–J).

### The protective effects of NRBF2 on endoplasmic reticulum stress-associated neuroinflammation and oxidative stress occurred via interaction with Rab7

**Fig. 5:**
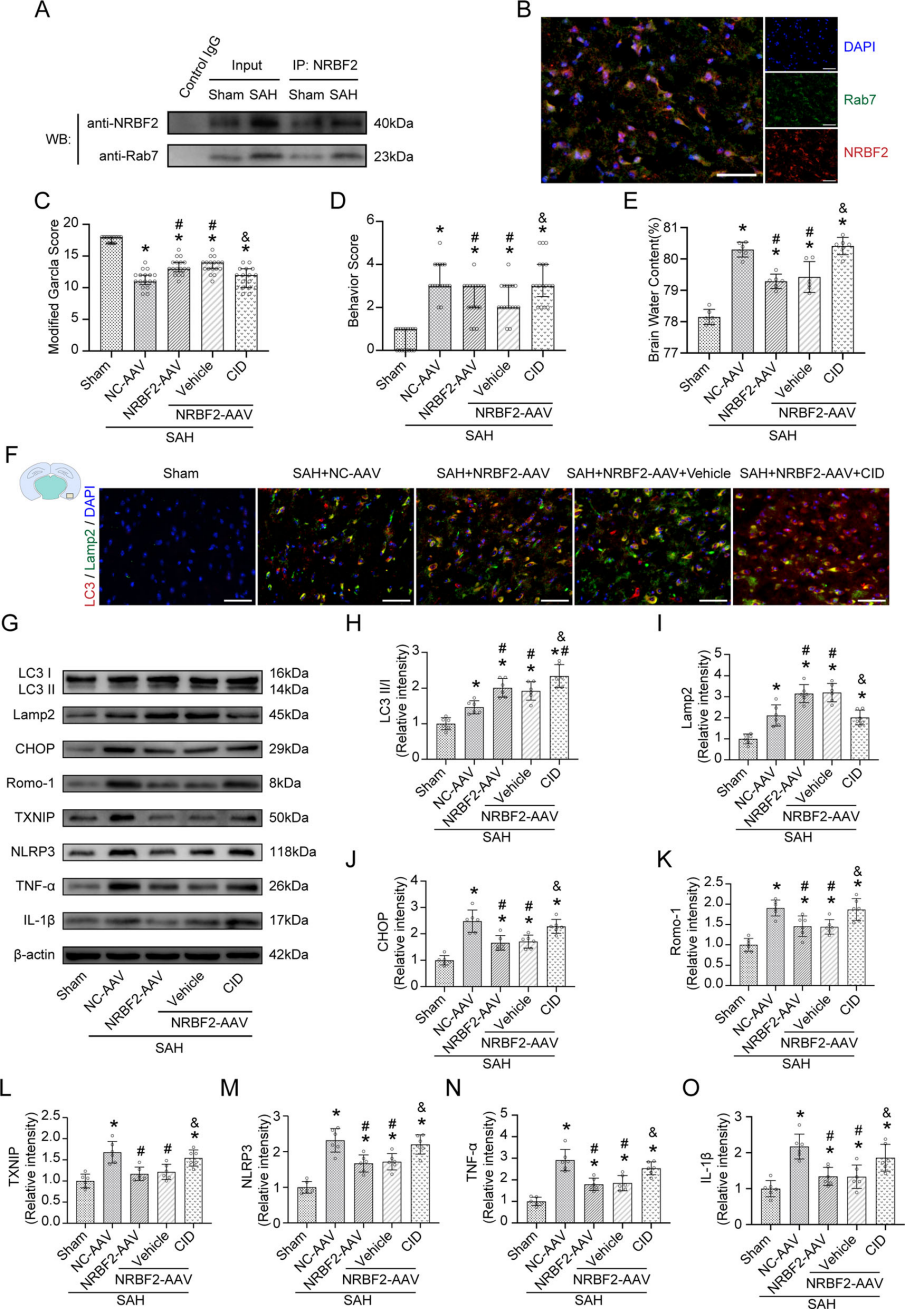
Pharmacological inhibition of Rab7 abolished the neuroprotective effect of NRBF2 against SAH at 24 h. **A** The lysates from cortex tissue were immunoprecipitated with anti-NRBF2. Then, immunoprecipitates were analyzed by western blotting with anti-NRBF2 and anti-Rab7. *n* = 6. **B** Immunostaining for NRBF2 and Rab7 in left cortex area. *n* = 3. **C**, **D** Quantification of neurological function with two different scoring systems at 24 after SAH. *n* = 17. **E** Quantification of brain water content. *n* = 6. **F** Representative microphotographs of immunofluorescence double staining showing the localization of LC3B (red) and Lamp2 (green). Scale bar = 50 μm. *n* = 5. (G-O) Representative western blotting images and quantitative analyses of LC3 II/I, Lamp2, CHOP, Romo-1, TXNIP, NLRP3, TNF-α, and IL-1β expression at 24 h after SAH. *n* = 6. Data are represented as mean ± SD (**E**, **H**–**O**) or median (interquartile range) (**C**, **D**). *P < 0.05 versus sham group. #*P* < 0.05 versus SAH + NC-AAV group. &*P* < 0.05 versus SAH+NRBF2-AAV + vehicle group

It has been reported that engagement of NRBF2 leads to recruitment and stimulation of Rab7 in modulating autophagosome maturation and then delivers an activation signal to downstream pathways [14]. We examined whether an association existed between NRBF2 and Rab7. By co-immunoprecipitation, NRBF2 appeared to interact with Rab7 in vivo (Fig. 5A). Co-immunolabeling further revealed that NRBF2 was co-localized with Rab7, rendering a physical basis for their interaction (Fig. 5B).

Compared with the control groups (SAH + NC-AAV group), the group treated with the Rab7 inhibitor CID abated the beneficial effects caused by NRBF2 overexpression on neurological scores and cerebral edema (Fig. 5C–E). Co-immunolabeling and western blotting analysis showed that NRBF2 overexpression evidently increased the expression of LC3 and Lamp2, whereas CID injection reversed the expression of Lamp2 but further increased the expression of LC3 (Fig. 5F–I). Additionally, CID injection terminated the beneficial effects caused by NRBF2 overexpression, resulting in increased expression of CHOP, Romo-1, TXNIP, NLRP3, TNF-α, and IL-1β (Fig. 5G, J–O).

### The MIT domain of NRBF2 is indispensable for the interaction between NRBF2 and Rab7

**Fig. 6:**
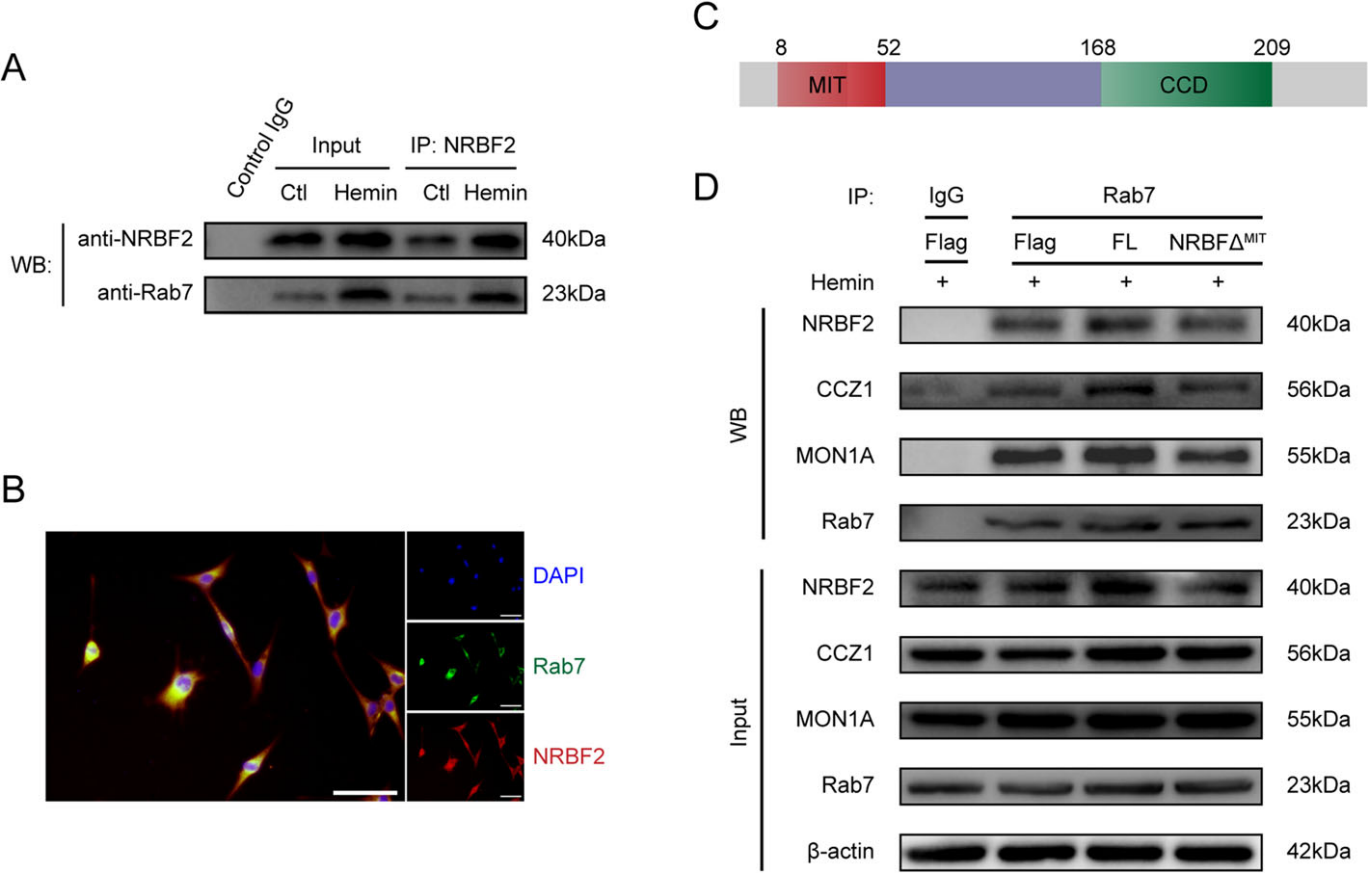
NRBF2 interacted with Rab7 and the MIT domain was indispensable for the interaction between NRBF2 and Rab7. **A** The lysates from HT22 cells were immunoprecipitated with anti-NRBF2. Then, immunoprecipitates were analyzed by western blotting with anti-NRBF2 and anti-Rab7. *n* = 5. **B** Immunostaining for NRBF2 and Rab7 in HT22 cells. *n* = 3. **C** Schematic diagram of NRBF2. **D** Cell lysates were immunoprecipitated with an anti-Rab7 antibody and subjected to western blotting with the indicated antibodies, including anti-NRBF2, -CCZ1, -MON1A, and -Rab7. *n* = 5

We first adopted HT22 cells, a cell line from mouse hippocampal neurons, to confirm that NRBF2 interacted with Rab7 in vitro by co-IP (Fig. 6A). Co-immunolabeling further revealed that NRBF2 was co-localized with Rab7 in HT22 cells, rendering a physical basis for their interaction (Fig. 6B).

NRBF2 contains an N-terminal MIT domain and the C-terminal central coiled-coil domain (CCD) (Fig. 6C) [9]. To further identify the critical domain of NRBF2 that interacts with Rab7, a mutant NRBF2 protein with a deleted MIT domain was constructed for binding studies. In contrast to full-length NRBF2, the data showed that the NRBF2^ΔMIT^ mutant alone lost the ability to bind to Rab7 under hemin stimulation (Fig. 6D). We speculated that the MIT domain was indispensable for full-length NRBF2 to compete with Rab7 binding for autophagy maturation. Indeed, we observed that NRBF2 overexpression dramatically enhanced the interaction of Rab7 with CCZ1 and MON1A, markers of autophagosome maturation (Fig. 6D). Conversely, the NRBF2^ΔMIT^ mutant exhibited limited effects compared with full-length NRBF2 (Fig. 6D). These data indicated that NRBF2 promoted CCZ1-MON1A-Rab7 complex assembly followed by autolysosome maturation. Moreover, the process was dependent on the MIT domain.

## Discussion

**Fig. 7:**
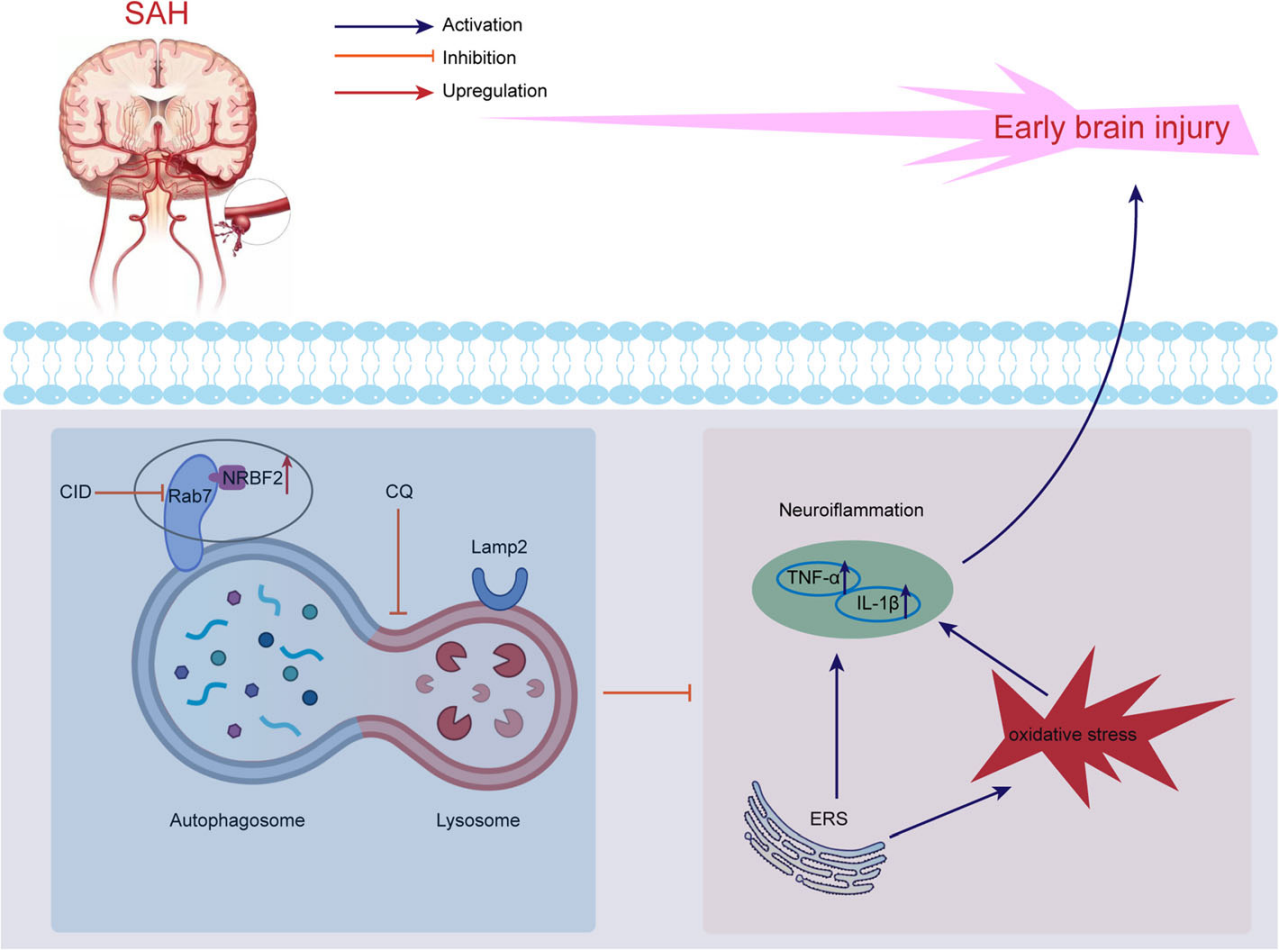
The potential molecular mechanisms of NRBF2-mediated ERS-associated neuroinflammation and oxidative stress via promoting autophagosome maturation by interacting with Rab7 after SAH

In our study, we found that the expression of NRBF2 was notably increased after SAH and peaked at 24 h after SAH. Upregulation of NRBF2 increased autophagy, and ameliorated ERS-associated neuroinflammation and oxidative stress, thus alleviating the neurological deficits and brain edema, whereas downregulation of NRBF2 reversed the above protective effects. Importantly, inhibition of the fusion between autophagosomes and lysosomes with CQ reversed the protective effect of NRBF2 on ERS-associated neuroinflammation and oxidative stress. Additionally, Rab7 was demonstrated to interact with NRBF2, and inhibiting Rab7 abolished the favorable effects of NRBF2 on the improvement of brain injury, regulation of autophagy maturation, and ERS-associated neuroinflammation and oxidative stress. Furthermore, the MIT domain was identified as indispensable for the interaction between NRBF2 and Rab7. Taken together, our findings indicate that NRBF2 conveys neuroprotection by ameliorating ERS-associated neuroinflammation and oxidative stress by promoting autophagosome maturation after SAH, at least in part through its interaction with Rab7 (Fig. 7).

More and more studies show the important role of ERS mediated neuroinflammation and oxidative stress in EBI after SAH [6, 7]. The accumulation of unfolded proteins in the endoplasmic reticulum represents a cellular stress induced by multiple stimuli and pathological conditions, which include hypoxia, oxidative injury, hypoglycemia, protein inclusion bodies, and viral infection [28, 29]. Pro-inflammatory cytokines, ROS, and dysfunctional organelles are produced in SAH, leading to unnormal protein folding, thereby activating endoplasmic reticulum stress and finally progressing to irreversible neurological deficits [6]. Afterwards, the overactivation of ERS aggravates the neuroinflammation and oxidative stress, including further microglia activation, which aggravates brain injury after stroke [6, 7, 30]. Therefore, inhibition of microglia activation and reduction of oxidative stress are beneficial to EBI after SAH.

Autophagy, a self-eating process, performs critical functions in SAH [4, 31]. Autophagy begins with the induction of autophagosome formation and ends with autophagosome degradation in lysosomes. Studies have shown that EBI, as a stress response mechanism, causes an increase in autophagosomes in SAH [32]. It is important to note that the activation of LC3 at a certain time point does not indicate autophagic flux. Changes in LC3 levels at a time point could only be explained by either increased autophagosome formation caused by activated autophagy or accumulated autophagosomes caused by autophagy dysfunction [33]. The formation of excessive ERS plays an important role in EBI-induced autophagic cell death after SAH [3–5]. Indeed, there was crosstalk among ERS, oxidative stress, and autophagy [34]. Previous studies have shown that both mitochondrial and endoplasmic reticulum fragments damaged by ROS are sequestered in autophagolysosomes to prevent leakage of calcium into the cytosol from these organelles [35]. Increasing studies have shown that crosstalk among ERS and autophagy might be the dawn of new therapeutic approaches [6, 36–38]. The role of the autophagy protein NRBF2 in our SAH study have also verified that enhanced autophagosome clearance is beneficial for neuroinflammation and oxidative stress by preventing overactivation of the ERS.

NRBF2 was newly identified as the fifth component of PtdIns3K complex 1 [39, 40]. Interestingly, we showed that the MIT domain of NRBF2 was important for regulating autophagy. In addition, the MIT domain was indispensable for the interaction between NRBF2 and Rab7 for autophagosome maturation. Previous studies showed that NRBF2 regulated autophagosome formation and maturation [9, 14]. As reported, NRBF2 could bind Atg14L directly and enhance Atg14L-linked Vps34 kinase activity and autophagy induction, and played a vital role in preventing ERS-mediated cytotoxicity and liver injury [9]. The effect of NRBF2 on the induction of autophagy was also confirmed by Cao et al. [39]. However, there is also a controversial study [41] suggested that the role of NRBF2 in regulating autophagy might be cell type dependent and needs further exploration. Ma et al. [12] reported that NRBF2, a critical molecular switch of PtdIns3K and autophagy activation, was controlled by phosphorylation via mTORC1. In Alzheimer’s disease, NRBF2 was identified to play a considerable role in regulating Alzheimer’s disease-associated protein degradation by modulating autophagy [11, 14]. In addition, NRBF2 may be a potential therapeutic target for cognitive impairment [13]. However, the role of NRBF2 in autophagosome maturation has only been explored in colitis and Alzheimer’s disease [10, 14]. This is the first study to explore the role of NRBF2 in SAH. According to our study, NRBF2 is a protective protein in injury of ERS-associated oxidative stress and neuroinflammation after SAH.

Rab7, found predominantly in late endosomes, has been recognized as the only lysosomal Rab protein in the Rab GTPase [42]. The key steps in the maturation of endosomes are the Rab5-Rab7 switch and the regulation of the MON1A-CCZ1 complex [43]. Rab7-mediated endosome maturation has been identified to be associated with several central nervous system diseases, such as Alzheimer’s disease, Parkinson’s disease, Huntington’s disease, and cerebral ischemic diseases [14, 44–46]. However, the role of Rab7 is also controversial, as previously reported. For instance, Zhan et al. [46] suggested that activation of Rab7 could enhance autophagosome maturation, presenting potential neuroprotection in ischemic rats, while Qin et al. [19] proposed that inhibition of Rab7 attenuated brain atrophy, improved neurologic function, and inhibited astrogliosis and glial scar formation after ischemic stroke. Therefore, the interplay between Rab7-induced autophagy maturation and stroke requires further exploration. Cai et al. showed that NRBF2 colocalized with Rab7 and was required for the generation of GTP-bound Rab7 by interacting with the Rab7 GEF CCZ1-MON1A and maintaining GEF activity [14]. Wu et al. showed that NRBF2 is necessary for the generation of the active form of Rab7 to promote fusion between phagosomes containing engulfed apoptotic cells and lysosomes by interacting with the MON1-CCZ1 complex and regulating the guanine nucleotide exchange factor (GEF) activity of the complex [10]. Our study is consistent with the above results that treatment with a Rab7 inhibitor reversed the anti-oxidative stress and anti-inflammation effects of NRBF2 in autophagosome maturation.

Research on the treatment of SAH-related injury has lasted for a long time, and the discovery of promising therapeutic targets has always been the goal of SAH researchers. NRBF2, explored in the current study, may become a promising therapeutic target for ERS-associated oxidative stress and neuroinflammation after SAH. It should be pointed out that the compensatory increase in NRBF2 in SAH itself is not enough. If intervention is carried out in SAH to make the increase in NRBF2 more obvious, it may play a better role in treatment. The clinical approach for NRBF2 intervention can be broken down into the following aspects: (1) research and development of new agonists; (2) exploration of existing clinical drugs with similar effects; (3) exploration of the upstream signaling pathway for NRBF2 to prepare for further clinical transformation. Although the present study verified the value of NRBF2 in a Rab7-relevant mechanism that mediated neuroprotection by promoting autophagosome maturation in the SAH model, some limitations of this study should not be ignored. First, it seems to be better to use a conditional knockout mouse model to exclude many background differences. Second, only the anti-inflammatory and anti-oxidative characteristics of the NRBF2 were evaluated in this study, without further investigation of its roles in apoptosis.

## Conclusions

This study shows that NRBF2 is upregulated at 24 h after SAH and exerts neuroprotection and attenuates EBI after SAH by reducing ERS-mediated neuroinflammation and oxidative stress by interacting with Rab7. These findings identify NRBF2-modulated autophagosome maturation as a potential target for alleviating EBI after SAH.

## Supplementary Information

**Additional file 1: Supplementary Figure S1.** Experimental design, animal groups and mortality.

**Additional file 2: Supplementary Figure S2.** Brain pictures and SAH grade. (A) Brains without or with SAH. (B) The quantification of SAH grade.

**Additional file 3: Supplementary Figure S3.** Effect of NRBF2 on long-term neurological function. (A, B) Escape latency and swimming distance of Morris water maze. n=10. (C) Representative swimming trajectories of the different groups in probe trials. (D) The crossovers of the platform location in the probe quadrant. n=10. (E) The percentage of time spent in the probe quadrant. n=10. Data are represented as mean ± SD. *P < 0.05 versus sham group. #*P* < 0.05 versus SAH group. &*P* < 0.05 versus SAH+Scr-siRNA group. @*P* < 0.05 versus SAH+NC-AAV group.

**Additional file 4: Supplementary Figure S4.** Autophagy was involved in the protective effect of NRBF2 after SAH. (A-C) Representative western blotting images and quantitative analyses of p62 and LC3 II/I expression at 24 h after SAH. n=6. (D, E) Quantification of neurological function with two different scoring systems at 24 after SAH. n=12. (F) Quantification of brain water content. n = 6. Data are represented as mean ± SD. *P < 0.05 versus sham group. #*P* < 0.05 versus SAH+NC-AAV group. &P < 0.05 versus SAH+NRBF2-AAV+vehicle1 group. (G-I) Representative western blotting images and quantitative analyses of p62 and LC3 II/I expression at 24 h after SAH. n=6. (J, K) Quantification of neurological function with two different scoring systems at 24 after SAH. n=12. (L) Quantification of brain water content. n = 6. Data are represented as mean ± SD (B, C, F, H, I, L) or median (interquartile range) (D, E, J, K). *P < 0.05 versus sham group. #*P* < 0.05 versus SAH+Scr-siRNA group. &*P* < 0.05 versus SAH+NRBF2-siRNA+vehicle2 group.

**Additional file 5: Supplementary Table S1.** Modified Garcia score.

**Additional file 6: Supplementary Table S2.** Behavioral Score.

**Additional file 7: Supplementary Table S3.** Physiological data of the mice after operation (dead mice are not included).

**Additional file 8: Supplementary Text S1.** Detailed procedures.

## Data Availability

The datasets analyzed during the current study are available from the corresponding author on reasonable request.

## Abbreviations

SAH: Subarachnoid hemorrhage
ERS: Endoplasmic reticulum stress
ROS: Reactive oxygen species
NRBF2: Nuclear receptor binding factor 2
IHC: Immunohistochemical
Scr: Scramble
NC: Negative control
BWC: Brain water content
TUNEL: Terminal deoxynucleotidyl transferase dUTP nick end labeling
IF: Immunofluorescence
DHE: Dihydroethidium
3-MA: 3-methyladenine
Rapa: Rapamycin
CQ: Chloroquine
Co-IP: Co-immunoprecipitation
CID: CID1067700
MWM: Morris water maze
FBS: Fetal bovine serum
BSA: Bovine serum albumin
DAB: 3,3-diaminobenzidine
SD: Standard deviation
ANOVA: One-way analysis of variance
AAV: Adeno-associated virus

## References

[1] Fan H, Ding R, Liu W, Zhang X, Li R, Wei B, Su S, Jin F, Wei C, He X (2021). Heat shock protein 22 modulates NRF1/TFAM-dependent mitochondrial biogenesis and DRP1-sparked mitochondrial apoptosis through AMPK-PGC1α signaling pathway to alleviate the early brain injury of subarachnoid hemorrhage in rats. Redox Biol.

[2] Macdonald RL, Schweizer TA (2017). Spontaneous subarachnoid haemorrhage. Lancet.

[3] Fan LF, He PY, Peng YC, Du QH, Ma YJ, Jin JX, Xu HZ, Li JR, Wang ZJ, Cao SL (2017). Mdivi-1 ameliorates early brain injury after subarachnoid hemorrhage via the suppression of inflammation-related blood-brain barrier disruption and endoplasmic reticulum stress-based apoptosis. Free Radic Biol Med.

[4] Galluzzi L, Bravo-San Pedro JM, Blomgren K, Kroemer G (2016). Autophagy in acute brain injury. Nat Rev Neurosci.

[5] Mo J, Enkhjargal B, Travis ZD, Zhou K, Wu P, Zhang G, Zhu Q, Zhang T, Peng J, Xu W, Ocak U, Chen Y, Tang J, Zhang J, Zhang JH (2019). AVE 0991 attenuates oxidative stress and neuronal apoptosis via Mas/PKA/CREB/UCP-2 pathway after subarachnoid hemorrhage in rats. Redox Biol.

[6] Xu W, Li T, Gao L, Zheng J, Yan J, Zhang J, Shao A (2019). Apelin-13/APJ system attenuates early brain injury via suppression of endoplasmic reticulum stress-associated TXNIP/NLRP3 inflammasome activation and oxidative stress in a AMPK-dependent manner after subarachnoid hemorrhage in rats. J Neuroinflammation.

[7] Zhao Q, Che X, Zhang H, Fan P, Tan G, Liu L, Jiang D, Zhao J, Xiang X, Liang Y, Sun X, He Z (2017). Thioredoxin-interacting protein links endoplasmic reticulum stress to inflammatory brain injury and apoptosis after subarachnoid haemorrhage. J Neuroinflammation.

[8] Zhang K, Kaufman RJ (2008). From endoplasmic-reticulum stress to the inflammatory response. Nature.

[9] Lu J, He L, Behrends C, Araki M, Araki K, Jun Wang Q, Catanzaro JM, Friedman SL, Zong WX, Fiel MI, Li M, Yue Z (2014). NRBF2 regulates autophagy and prevents liver injury by modulating Atg14L-linked phosphatidylinositol-3 kinase III activity. Nat Commun.

[10] Wu MY, Liu L, Wang EJ, Xiao HT, Cai CZ, Wang J, Su H, Wang Y, Tan J, Zhang Z, Wang J, Yao M, Ouyang DF, Yue Z, Li M, Chen Y, Bian ZX, Lu JH (2020). PI3KC3 complex subunit NRBF2 is required for apoptotic cell clearance to restrict intestinal inflammation. Autophagy.

[11] Yang C, Cai CZ, Song JX, Tan JQ, Durairajan SSK, Iyaswamy A, Wu MY, Chen LL, Yue Z, Li M, Lu JH (2017). NRBF2 is involved in the autophagic degradation process of APP-CTFs in Alzheimer disease models. Autophagy.

[12] Ma X, Zhang S, He L, Rong Y, Brier LW, Sun Q, Liu R, Fan W, Chen S, Yue Z, Kim J, Guan KL, Li D, Zhong Q (2017). MTORC1-mediated NRBF2 phosphorylation functions as a switch for the class III PtdIns3K and autophagy. Autophagy.

[13] Lachance V, Wang Q, Sweet E, Choi I, Cai CZ, Zhuang XX, Zhang Y, Jiang JL, Blitzer RD, Bozdagi-Gunal O, Zhang B, Lu JH, Yue Z (2019). Autophagy protein NRBF2 has reduced expression in Alzheimer's brains and modulates memory and amyloid-beta homeostasis in mice. Mol Neurodegener.

[14] Cai CZ, Yang C, Zhuang XX, Yuan NN, Wu MY, Tan JQ, Song JX, Cheung KH, Su H, Wang YT, Tang BS, Behrends C, Durairajan SSK, Yue Z, Li M, Lu JH (2020). NRBF2 is a RAB7 effector required for autophagosome maturation and mediates the association of APP-CTFs with active form of RAB7 for degradation. Autophagy.

[15] Sinha P, Verma B, Ganesh S (2021). Trehalose ameliorates seizure susceptibility in lafora disease mouse models by suppressing neuroinflammation and endoplasmic reticulum stress. Mol Neurobiol.

[16] Fujimoto M, Shiba M, Kawakita F, Liu L, Shimojo N, Imanaka-Yoshida K, Yoshida T, Suzuki H (2016). Deficiency of tenascin-C and attenuation of blood-brain barrier disruption following experimental subarachnoid hemorrhage in mice. J Neurosurg.

[17] Wu X, Zheng Y, Liu M, Li Y, Ma S, Tang W, Yan W, Cao M, Zheng W, Jiang L, Wu J, Han F, Qin Z, Fang L, Hu W, Chen Z, Zhang X (2020). BNIP3L/NIX degradation leads to mitophagy deficiency in ischemic brains. Autophagy.

[18] Yamamoto S, Mutoh T, Sasaki K, Mutoh T, Taki Y (2019). Central action of rapamycin on early ischemic injury and related cardiac depression following experimental subarachnoid hemorrhage. Brain Res Bull.

[19] Qin Y, He Y, Zhu YM, Li M, Ni Y, Liu J, Zhang HL (2019). CID1067700, a late endosome GTPase Rab7 receptor antagonist, attenuates brain atrophy, improves neurologic deficits and inhibits reactive astrogliosis in rat ischemic stroke. Acta Pharmacol Sin.

[20] Zhang X, Jing Y, Qin C, Liu C, Yang D, Gao F, Yang M, Du L, Li J (2020). Mechanical stress regulates autophagic flux to affect apoptosis after spinal cord injury. J Cell Mol Med.

[21] Suzuki H, Zhang JH, Chen J, Xu XM, Xu CZ, Zhang JH (2012). Neurobehavioral assessments of subarachnoid hemorrhage. Animal Models of Acute Neurological Injuries II: Injury and Mechanistic Assessments.

[22] Suzuki H, Hasegawa Y, Kanamaru K, Zhang JH (2010). Mechanisms of osteopontin-induced stabilization of blood-brain barrier disruption after subarachnoid hemorrhage in rats. Stroke.

[23] Peng Y, Zhuang J, Ying G, Zeng H, Zhou H, Cao Y, Chen H, Xu C, Fu X, Xu H, Li J, Cao S, Chen J, Gu C, Yan F, Chen G (2020). Stimulator of IFN genes mediates neuroinflammatory injury by suppressing AMPK signal in experimental subarachnoid hemorrhage. J Neuroinflammation.

[24] Cao Y, Li Y, He C, Yan F, Li JR, Xu HZ, Zhuang JF, Zhou H, Peng YC, Fu XJ, Lu XY, Yao Y, Wei YY, Tong Y, Zhou YF, Wang L (2021). Selective Ferroptosis Inhibitor Liproxstatin-1 Attenuates Neurological Deficits and Neuroinflammation After Subarachnoid Hemorrhage. Neurosci Bull.

[25] Zhuang J, Peng Y, Gu C, Chen H, Lin Z, Zhou H, Wu X, Li J, Yu X, Cao Y, Zeng H, Fu X, Xu C, Huang P, Cao S, Wang C, Yan F, Chen G (2020). Wogonin accelerates hematoma clearance and improves neurological outcome via the PPAR-γ pathway after intracerebral hemorrhage. Transl Stroke Res.

[26] Xu W, Yan J, Ocak U, Lenahan C, Shao A, Tang J, Zhang J, Zhang JH (2021). Melanocortin 1 receptor attenuates early brain injury following subarachnoid hemorrhage by controlling mitochondrial metabolism via AMPK/SIRT1/PGC-1α pathway in rats. Theranostics.

[27] Xu P, Zhang X, Liu Q, Xie Y, Shi X, Chen J, Li Y, Guo H, Sun R, Hong Y, Liu X, Xu G (2019). Microglial TREM-1 receptor mediates neuroinflammatory injury via interaction with SYK in experimental ischemic stroke. Cell Death Dis.

[28] Kim I, Xu W, Reed JC (2008). Cell death and endoplasmic reticulum stress: disease relevance and therapeutic opportunities. Nat Rev Drug Discov.

[29] Ren J, Bi Y, Sowers JR, Hetz C, Zhang Y (2021). Endoplasmic reticulum stress and unfolded protein response in cardiovascular diseases. Nat Rev Cardiol.

[30] Sehba FA, Hou J, Pluta RM, Zhang JH (2012). The importance of early brain injury after subarachnoid hemorrhage. Prog Neurobiol.

[31] Chen J, Wang L, Wu C, Hu Q, Gu C, Yan F, Li J, Yan W, Chen G (2014). Melatonin-enhanced autophagy protects against neural apoptosis via a mitochondrial pathway in early brain injury following a subarachnoid hemorrhage. J Pineal Res.

[32] Lee JY, He Y, Sagher O, Keep R, Hua Y, Xi G (2009). Activated autophagy pathway in experimental subarachnoid hemorrhage. Brain Res.

[33] Li Y, Liang P, Jiang B, Tang Y, Liu X, Liu M, Sun H, Chen C, Hao H, Liu Z, Xiao X (2020). CARD9 promotes autophagy in cardiomyocytes in myocardial ischemia/reperfusion injury via interacting with Rubicon directly. Basic Res Cardiol.

[34] Nakka VP, Prakash-Babu P, Vemuganti R (2016). Crosstalk Between Endoplasmic Reticulum Stress, Oxidative Stress, and Autophagy: Potential Therapeutic Targets for Acute CNS Injuries. Mol Neurobiol.

[35] Ding WX, Ni HM, Gao W, Yoshimori T, Stolz DB, Ron D, Yin XM (2007). Linking of autophagy to ubiquitin-proteasome system is important for the regulation of endoplasmic reticulum stress and cell viability. Am J Pathol.

[36] Cui J, Liu Y, Chang X, Gou W, Zhou X, Liu Z, Li Z, Wu Y, Zuo D (2019). Acetaldehyde Induces Neurotoxicity In Vitro via Oxidative Stress- and Ca(2+) Imbalance-Mediated Endoplasmic Reticulum Stress. Oxid Med Cell Longev.

[37] Liu S, Xin D, Wang L, Zhang T, Bai X, Li T, Xie Y, Xue H, Bo S, Liu D, Wang Z (2017). Therapeutic effects of L-Cysteine in newborn mice subjected to hypoxia-ischemia brain injury via the CBS/H(2)S system: role of oxidative stress and endoplasmic reticulum stress. Redox Biol.

[38] Yang Y, White E (2020). Autophagy suppresses TRP53/p53 and oxidative stress to enable mammalian survival. Autophagy.

[39] Cao Y, Wang Y, Abi Saab WF, Yang F, Pessin JE, Backer JM (2014). NRBF2 regulates macroautophagy as a component of Vps34 Complex I. Biochem J.

[40] Young LN, Cho K, Lawrence R, Zoncu R, Hurley JH (2016). Dynamics and architecture of the NRBF2-containing phosphatidylinositol 3-kinase complex I of autophagy. Proc Natl Acad Sci U S A.

[41] Zhong Y, Morris DH, Jin L, Patel MS, Karunakaran SK, Fu YJ, Matuszak EA, Weiss HL, Chait BT, Wang QJ (2014). Nrbf2 protein suppresses autophagy by modulating Atg14L protein-containing Beclin 1-Vps34 complex architecture and reducing intracellular phosphatidylinositol-3 phosphate levels. J Biol Chem.

[42] Hyttinen JM, Niittykoski M, Salminen A, Kaarniranta K (1833). Maturation of autophagosomes and endosomes: a key role for Rab7. Biochim Biophys Acta.

[43] Wen H, Zhan L, Chen S, Long L, Xu E (2017). Rab7 may be a novel therapeutic target for neurologic diseases as a key regulator in autophagy. J Neurosci Res.

[44] Saridaki T, Nippold M, Dinter E, Roos A, Diederichs L, Fensky L, Schulz JB, Falkenburger BH (2018). FYCO1 mediates clearance of α-synuclein aggregates through a Rab7-dependent mechanism. J Neurochem.

[45] White JA, Anderson E, Zimmerman K, Zheng KH, Rouhani R, Gunawardena S (2015). Huntingtin differentially regulates the axonal transport of a sub-set of Rab-containing vesicles in vivo. Hum Mol Genet.

[46] Zhan L, Chen S, Li K, Liang D, Zhu X, Liu L, Lu Z, Sun W, Xu E (2017). Autophagosome maturation mediated by Rab7 contributes to neuroprotection of hypoxic preconditioning against global cerebral ischemia in rats. Cell Death Dis.

