# Supplementary figures and images for: Autophagy protein NRBF2 attenuates endoplasmic reticulum stress-associated neuroinflammation and oxidative stress via promoting autophagosome maturation by interacting with Rab7 after SAH
Source: J Neuroinflammation. 2021 Sep 16;18:210. doi: 10.1186/s12974-021-02270-4 (PMC8447596; doi:10.1186/s12974-021-02270-4)

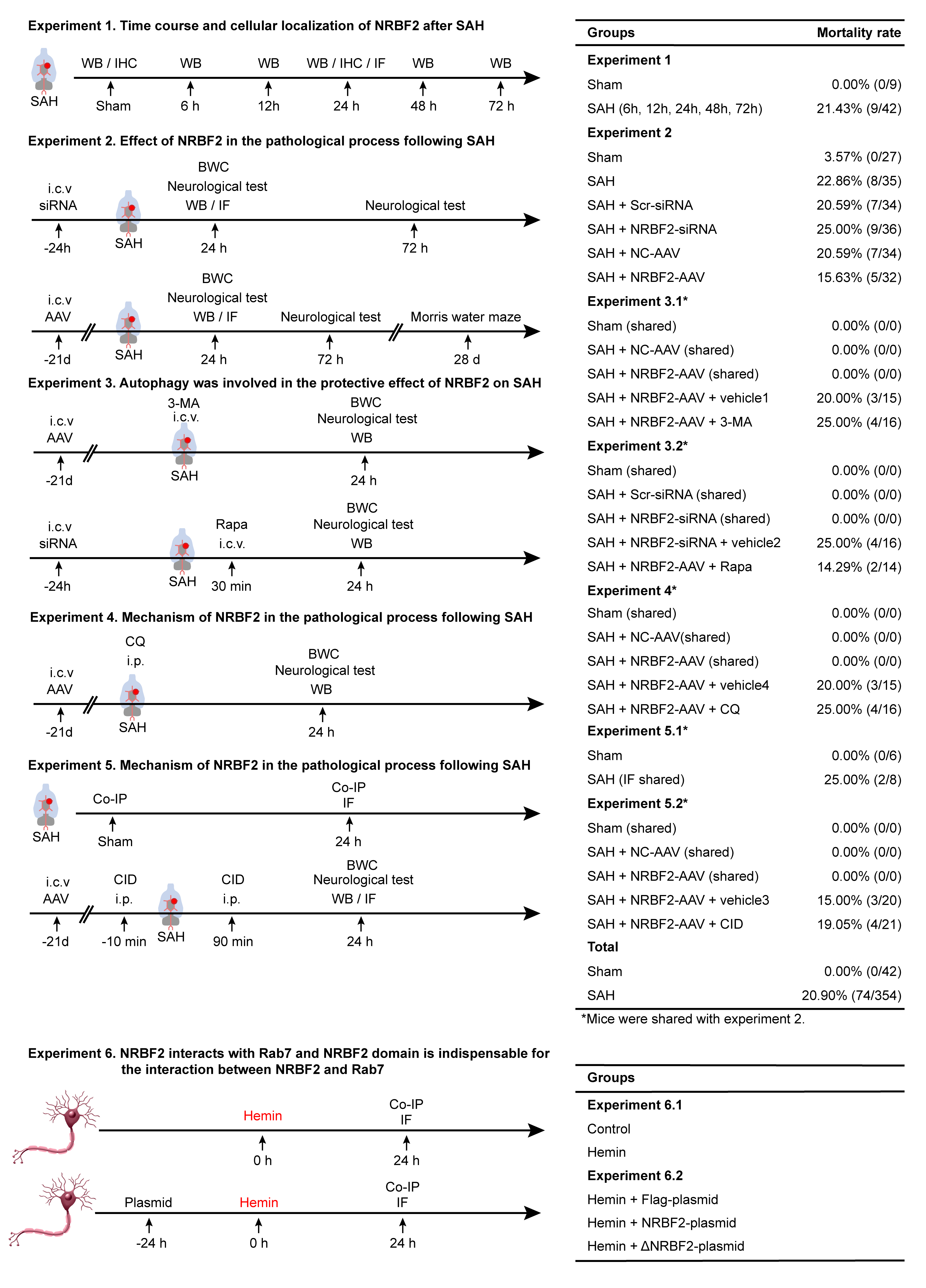

Supplement: Supplementary file 1 — Additional file 1: Supplementary Figure S1. Experimental design, animal groups and mortality. [file 12974_2021_2270_MOESM1_ESM.tif]

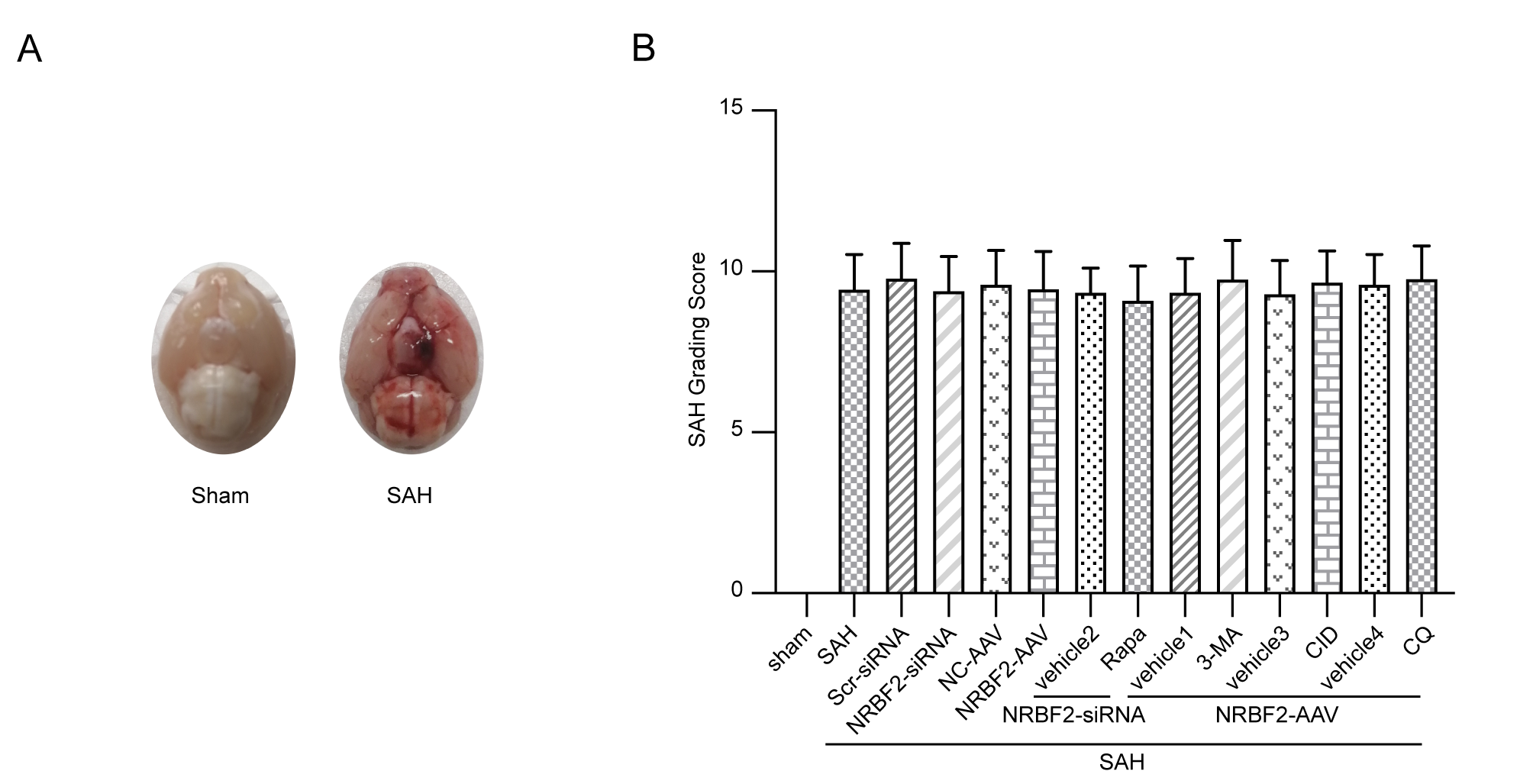

Supplement: Supplementary file 2 — Additional file 2: Supplementary Figure S2. Brain pictures and SAH grade. (A) Brains without or with SAH. (B) The quantification of SAH grade. [file 12974_2021_2270_MOESM2_ESM.tif]

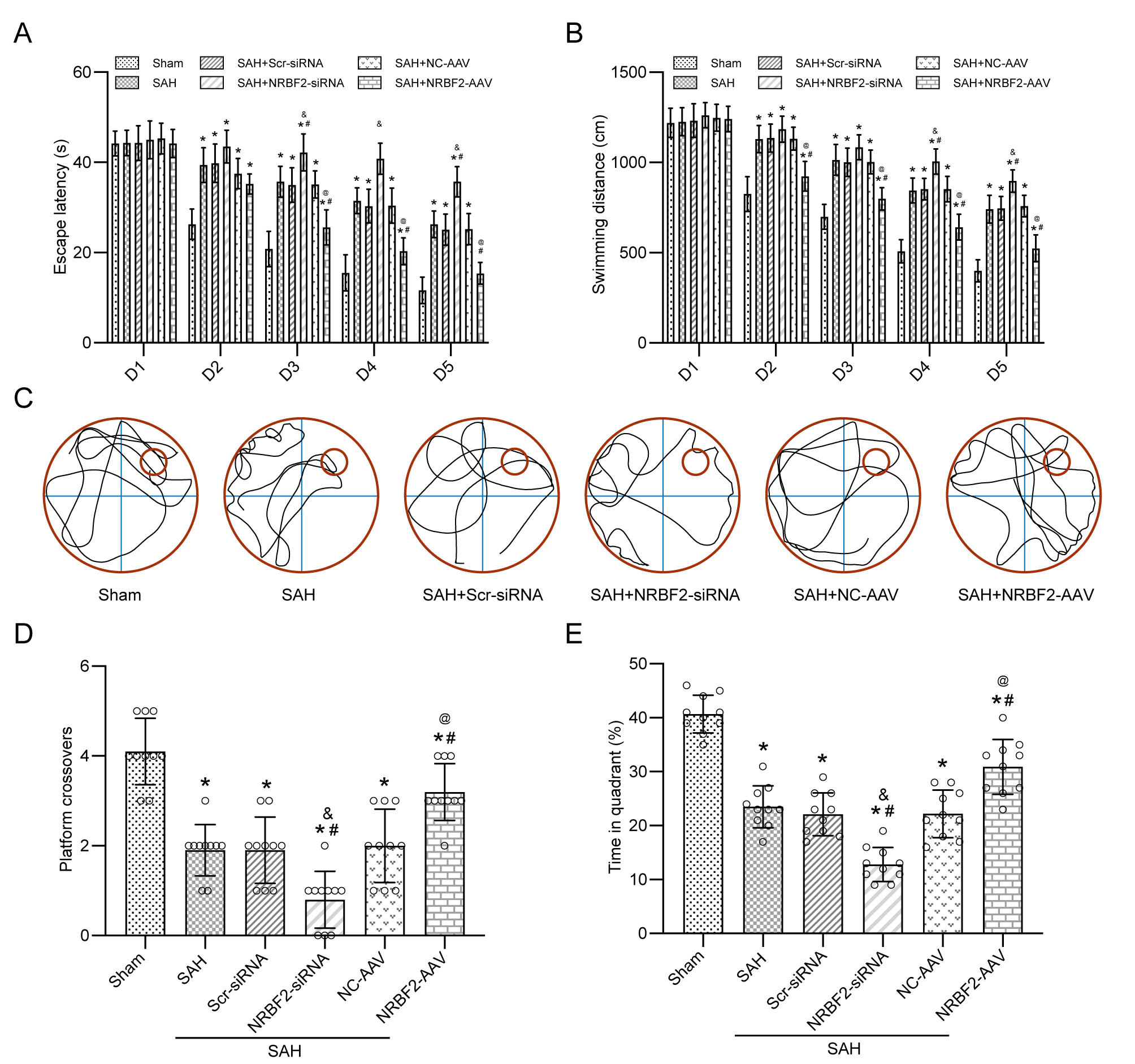

Supplement: Supplementary file 3 — Additional file 3: Supplementary Figure S3. Effect of NRBF2 on long-term neurological function. (A, B) Escape latency and swimming distance of Morris water maze. n=10. (C) Representative swimming trajectories of the different groups in probe trials. (D) The crossovers of the platform location in the probe quadrant. n=10. (E) The percentage of time spent in the probe quadrant. n=10. Data are represented as mean ± SD. *P < 0.05 versus sham group. #P < 0.05 versus SAH group. &P < 0.05 versus SAH+Scr-siRNA group. @P < 0.05 versus SAH+NC-AAV group. [file 12974_2021_2270_MOESM3_ESM.tif]

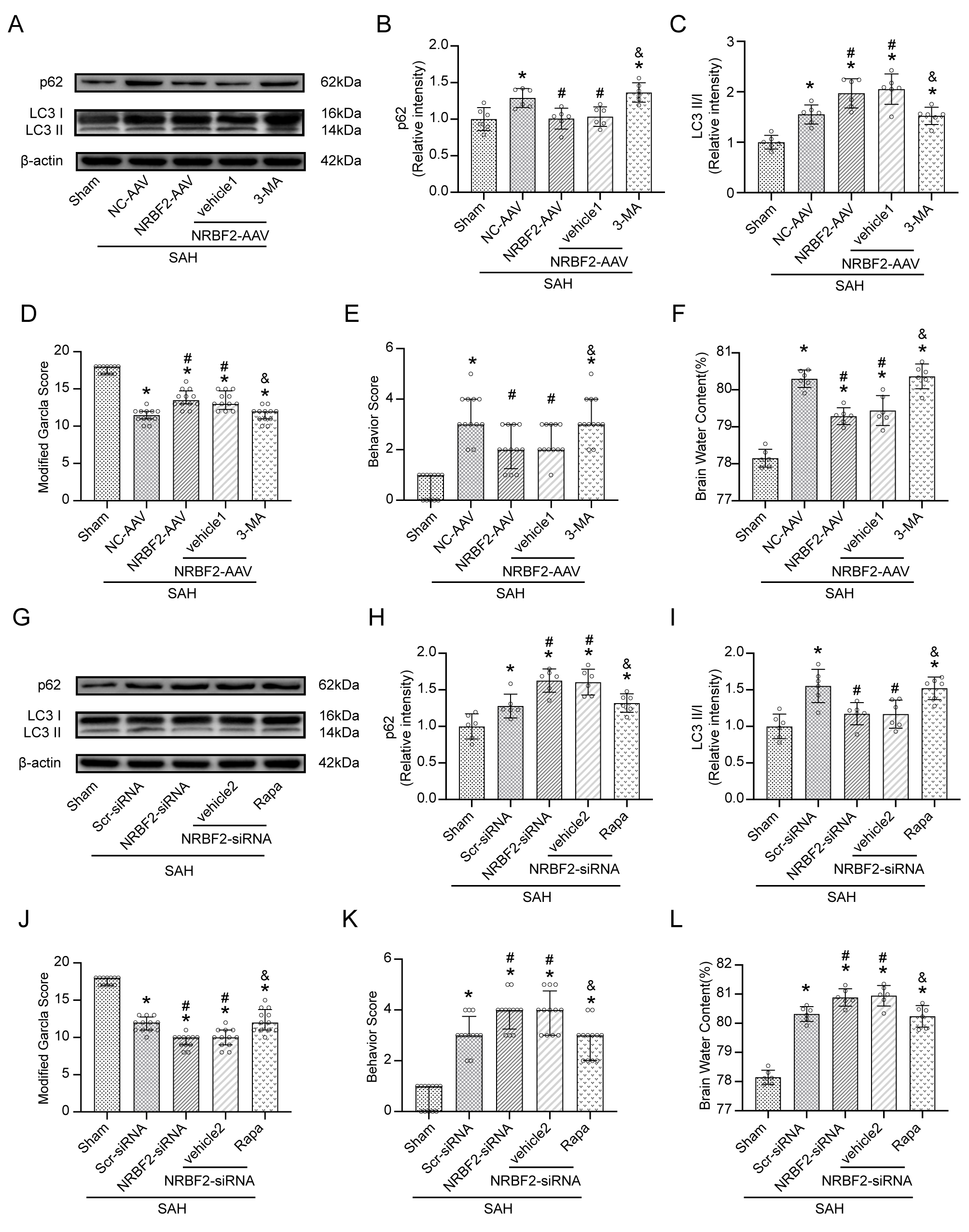

Supplement: Supplementary file 4 — Additional file 4: Supplementary Figure S4. Autophagy was involved in the protective effect of NRBF2 after SAH. (A-C) Representative western blotting images and quantitative analyses of p62 and LC3 II/I expression at 24 h after SAH. n=6. (D, E) Quantification of neurological function with two different scoring systems at 24 after SAH. n=12. (F) Quantification of brain water content. n = 6. Data are represented as mean ± SD. *P < 0.05 versus sham group. #P < 0.05 versus SAH+NC-AAV group. &P < 0.05 versus SAH+NRBF2-AAV+vehicle1 group. (G-I) Representative western blotting images and quantitative analyses of p62 and LC3 II/I expression at 24 h after SAH. n=6. (J, K) Quantification of neurological function with two different scoring systems at 24 after SAH. n=12. (L) Quantification of brain water content. n = 6. Data are represented as mean ± SD (B, C, F, H, I, L) or median (interquartile range) (D, E, J, K). *P < 0.05 versus sham group. #P < 0.05 versus SAH+Scr-siRNA group. &P < 0.05 versus SAH+NRBF2-siRNA+vehicle2 group. [file 12974_2021_2270_MOESM4_ESM.tif]
