# Supplementary material for: Autophagy protein NRBF2 attenuates endoplasmic reticulum stress-associated neuroinflammation and oxidative stress via promoting autophagosome maturation by interacting with Rab7 after SAH
Source: J Neuroinflammation. 2021 Sep 16;18:210. doi: 10.1186/s12974-021-02270-4 (PMC8447596; doi:10.1186/s12974-021-02270-4)
Supplement: Supplementary file 5 — Additional file 5: Supplementary Table S1. Modified Garcia score. [file 12974_2021_2270_MOESM5_ESM.docx]

**Supplementary Table S1**. Modified Garcia score.

| Item | Behavior | Score |
| --- | --- | --- |
| Spontaneous Activity (in cage for 5 min)  Spontaneous movements of all limbs  Movements of forelimbs  Climbing wall of wire cage  Reaction to touch on both side of trunk  Response to vibrissae touch | No movement  Barely moves position  Moves but does not approach at least three sides of cage  Moves and approaches at least three sides of cage  No movement  Slight movement of limbs  Moves all limbs but slowly  Move all limbs same as pre-SAH  No outreaching  Slight outreaching  Outreach is limited and less than pre-SAH  Outreach same as pre-SAH  Fails to climb  Climbs weakly  Normal climbing  No response  Weak response  Normal response  No response  Weak response  Normal response | 0  1  2  3  0  1  2  3  0  1  2  3  1  2  3  1  2  3  1  2  3 |
