# Supplementary material for: Autophagy protein NRBF2 attenuates endoplasmic reticulum stress-associated neuroinflammation and oxidative stress via promoting autophagosome maturation by interacting with Rab7 after SAH
Source: J Neuroinflammation. 2021 Sep 16;18:210. doi: 10.1186/s12974-021-02270-4 (PMC8447596; doi:10.1186/s12974-021-02270-4)
Supplement: Supplementary file 6 — Additional file 6: Supplementary Table S2. Behavioral Score. [file 12974_2021_2270_MOESM6_ESM.docx]

**Supplementary Table S2**. Behavioral Score.

| Item | Behavior | Score |
| --- | --- | --- |
| Appetite | Finished meal | 0 |
|  | Left meal unfinished | 1 |
|  | Scarcely ate | 2 |
| Activity | Active, squeaking or standing | 0 |
|  | Lying down, will stand, and walk with some stimulation | 1 |
|  | Almost always lying down | 2 |
| Deficits | No deficits | 0 |
|  | Unable to walk because of ataxia or paresis | 1 |
|  | Impossible to walk and stand because of ataxia and paresis | 2 |
