# Supplementary material for: Autophagy protein NRBF2 attenuates endoplasmic reticulum stress-associated neuroinflammation and oxidative stress via promoting autophagosome maturation by interacting with Rab7 after SAH
Source: J Neuroinflammation. 2021 Sep 16;18:210. doi: 10.1186/s12974-021-02270-4 (PMC8447596; doi:10.1186/s12974-021-02270-4)
Supplement: Supplementary file 7 — Additional file 7: Supplementary Table S3. Physiological data of the mice after operation (dead mice are not included). [file 12974_2021_2270_MOESM7_ESM.docx]

**Supplementary Table S3**. **Physiological data of the mice after operation (dead mice are not included).**

| Group | Mice used | | MAP (mmHg) | Arterial pH | PO2 (mmHg) | PCO2 (mmHg) | Blood glucose (mg/dl) |
| --- | --- | --- | --- | --- | --- | --- | --- |
| sham  SAH  SAH + Scr-siRNA  SAH + NRBF2-siRNA  SAH + NC-AAV  SAH + NRBF2-AAV  SAH + NRBF2-AAV + vehicle 1  SAH + NRBF2-AAV + 3-MA  SAH + NRBF2-siRNA + vehicle 2  SAH + NRBF2-siRNA + Rapa  SAH + NRBF2-AAV + vehicle 3  SAH + NRBF2-AAV + CID  SAH + NRBF2-AAV + vehicle 4  SAH + NRBF2-AAV + CQ | 42  66  27  27  27  27  12  12  12  12  17  17  17  17 | 108±6  109±5  109±8  111±5  110±9  111±6  111±4  112±7  110±7  113±5  114±6  107±8  111±8  106±5 | | 7.39±0.21  7.39±0.32  7.39±0.17  7.39±0.29  7.39±0.11  7.37±0.15  7.41±0.12  7.38±0.10  7.39±0.18  7.40±0.17  7.41±0.19  7.39±0.09  7.42±0.08  7.38±0.14 | 95.8±1.9  94.3±2.7  95.4±2.0  93.4±3.1  95.4±3.2  94.9±2.2  94.1±1.7  94.6±1.9  95.1±2.6  95.5±2.0  95.1±1.4  95.6±1.6  94.1±1.8  95.8±2.6 | 37.9±1.1  36.4±1.7  37.3±2.1  35.6±1.5  37.0±2.5  36.0±1.2  36.9±1.9  36.8±2.2  37.4±1.8  37.0±2.4  37.2±1.5  36.2±1.9  37.6±1.3  36.0±2.2 | 121±22  132±20  113±25  115±29  109±18  123±14  120±25  123±19  119±22  124±21  121±15  133±21  118±19  131±28 |
