# Supplementary material for: Autophagy protein NRBF2 attenuates endoplasmic reticulum stress-associated neuroinflammation and oxidative stress via promoting autophagosome maturation by interacting with Rab7 after SAH
Source: J Neuroinflammation. 2021 Sep 16;18:210. doi: 10.1186/s12974-021-02270-4 (PMC8447596; doi:10.1186/s12974-021-02270-4)
Supplement: Supplementary file 8 — Additional file 8: Supplementary Text S1. Detailed procedures. [file 12974_2021_2270_MOESM8_ESM.docx]

**Supplementary Text S1.** Detailed procedures.

SAH animal model

The mice were anesthetized using 2% isoflurane and ventilated with 1% isoflurane. Mice were closely monitored physiological parameters throughout the procedure. Briefly, after exposing the left carotid artery and its branches, a 5–0 sharpened monofilament nylon suture was advanced and finally reached the bifurcation of the anterior and middle cerebral artery. Then, vessel perforation was executed to produce SAH. The sham-operated mice underwent the same surgical procedure but without vessel perforation.

Intracerebroventricular injection

The mice were anesthetized with 2% isoflurane and ventilated with 1% isoflurane as described above. Next, we used a drill to make a small burr hole 1 mm posterior to the bregma and 1.0 mm right lateral to the midline, at which point the drug was slowly administered (3.2 mm in depth, 0.5 μl/min). The needle was kept in place for 5 minutes, and then was slowly withdrawn over a period of 5 minutes. Finally, the burr hole and incision were closed with bone wax and sutures, respectively. All surgical procedures were conducted with sterile techniques.
